# Reshuffling and Relocating: The Gendered and Income-Related Differential Effects of Restricting Smoking Locations

**DOI:** 10.1155/2012/907832

**Published:** 2012-04-24

**Authors:** Natalie Hemsing, Lorraine Greaves, Nancy Poole, Joan Bottorff

**Affiliations:** ^1^British Columbia Centre of Excellence for Women's Health, E311-4500 Oak Street (Box 48), Vancouver, BC, Canada V6H 3N1; ^2^School of Nursing, University of British Columbia, 3333 University Way, Kelowna, BC, Canada V6T 1Z3

## Abstract

This study investigates secondhand smoke (SHS) exposure and management in the context of smoking location restrictions, for nonsmokers, former, and current smokers. A purposive sample of 47 low income and non-low-income men and women of varied smoking statuses was recruited to participate in a telephone interview or a focus group. Amidst general approval of increased restrictions there were gendered patterns of SHS exposure and management, and effects of SHS policies that reflect power, control, and social roles that need to be considered as policies are developed, implemented and monitored. The experience of smoking restrictions and the management of SHS is influenced by the social context (relationship with a partner, family member, or stranger), the space of exposure (public or private, worksite), the social location of individuals involved (gender, income), and differential tolerance to SHS. This confluence of factors creates differing unintended and unexpected consequences to the social and physical situations of male and female smokers, nonsmokers, and former smokers. These factors deserve further study, in the interests of informing the development of future interventions and policies restricting SHS.

## 1. Introduction

Smoking restrictions in public places, or secondhand smoke (SHS) policies, are increasingly common in many parts of the world. In some countries, smoking restrictions have extended to private spaces (such as cars) and outdoor spaces (such as doorways, patios, parks, and beaches). In the province of British Columbia, Canada, smoking restrictions exist in workplaces including restaurants and bars (since 2001 in some municipalities), within 3 metres from doorways of public buildings (since 2008), and in cars where a person under the age of 16 is present (since April 2009). Several municipalities have even stronger bylaws. In Vancouver, for example, smoking is prohibited on restaurant/bar patios, within 6 metres of doorways, at bus shelters, and on beaches and parks [1]. 

The potential health effects of SHS have been widely documented as support for these policies in Canada [2], including the effect of SHS exposure in increasing the risk for cancer, heart disease, and lung diseases [3, 4]. Some research has concluded that smoking restrictions are associated with a decrease in SHS exposure (Callinan, Clarke et al. [5]), [6, 7] and may also be associated with increased smoking reduction or cessation [8–10]. SHS policies have the potential to improve health and decrease risk for disease [11].

However, the gendered implications of SHS initiatives and policies for women and men may result in specific unintended consequences as gendered dynamics shape women's and men's smoking behaviours, place of exposure, and management of SHS. For example, women may confront challenges in managing SHS exposure in the home due to gender inequalities within the domestic sphere [12]. Women and men may also face specific vulnerabilities due to the gendered and classed nature of work and the type of jobs women and men living on a low income are more likely to occupy. For example, one focus group study found that low-income women working primarily in office and retail environments reported a prosmoking environment (including more opportunities for smoke breaks and the presence of other coworkers who smoke) [13]. Low-income men, particularly older men working in outdoor environments, also noted a prosmoking context and lack of smoking restrictions [13]. Another study found that smoking restrictions in bars and restaurants in California have resulted in more smokers congregating in bars where restrictions are not enforced, where low-income women are typically employed, thereby increasing their vulnerability to SHS exposure [14].

Research also suggests that women and men of different income levels may encounter specific vulnerabilities due to social and physical disadvantage. Residing in a low-income area [15, 16] or an area of physical disorder or deprivation [17] has been linked to greater tobacco use. In a qualitative study exploring the social context of smoking, participants in low-income groups reported that they had not perceived a decline in smoking, and often described smoking as being more socially acceptable in low-income neighbourhoods [13]. A review of the unintended consequences of SHS policies for disadvantaged women revealed that women living on a low income may experience more barriers to quitting smoking and more vulnerabilities to SHS exposure [18].

In short, social, economic, and environmental issues shape the conditions of women and men's smoking and exposure to SHS and their responses to smoking restrictions. This study explored the effects of SHS policies on diverse groups of women and men who smoke and/or are exposed to SHS, and gauges the relationship between their social and built environments and their capacity to manage SHS exposure. In particular, what are the consequences of location restrictions for women and men, and how do the social (i.e., social roles, social positions) and built environments (i.e., housing conditions, work facilities, and daily settings) that women and men experience and inhabit influence their capacity to manage smoke exposure? To explore these questions, we asked women and men to describe the context of their SHS exposure (source and setting, and challenges in managing exposure) and discuss their experiences of smoking restrictions in Vancouver.

## 2. Background

The majority of studies investigating SHS policies focus on the impact of various policies on health outcomes and public opinion following implementation. Many studies have examined the impact of SHS policies on smoking cessation in the workplace [19–28] and the home [29–33]. In addition, researchers have investigated attitudes and public support following implementation of SHS policies [34–37].

A body of literature also exists on the connection between smoking restrictions and the denormalization of smoking—the process wherein smoking has gradually been redefined as socially unacceptable [38, 39]. Several authors have discussed the potential for discrimination and stigma among smokers as an adverse outcome of location restrictions, particularly among already vulnerable populations [39–42]. There have been debates among researchers over whether denormalization and stigma are effective tobacco control strategies [43, 44] or unethical and discriminatory against smokers [45, 46]. More recently, debates have centred around smoking restrictions in outdoor spaces, with some researchers claiming these policies may be particularly stigmatizing and are backed by relatively weak scientific evidence of health harms [45, 47, 48]. Denormalization may actually impede cessation efforts, particularly for socially disadvantaged smokers. For example, Thompson and colleagues examined the effect of the denormalization of smoking and associated smoking-related stigma on creating “smoking islands” where smoking becomes normalized and smoking reduction and cessation efforts are inhibited [49].

Several studies have examined individual responses to SHS policies and SHS management. Bell and colleagues explored responses to location restrictions among smokers in Vancouver and found that smokers experienced stigma, and changes in their access to and use of space [50]. Ritchie and colleagues' qualitative study of stigma following SHS legislation in Scotland revealed that smokers utilized different strategies to cope with stigma, such as managing spaces where they smoke, limiting social activities, stigmatizing other smokers and/or discussing the benefits of location restrictions [51]. Their study of the social context of smoking in Scotland following legislation, also revealed changes in participants' use of public space and smoking behaviours following implementation [52]. Poland and colleagues identified heterogeneity in smokers and nonsmokers attitudes to SHS management, distinguishing between the various tolerance levels, interactions, and response styles of smokers and nonsmokers to smoke exposure [53]. Robinson and colleagues discovered a range of implementation styles among participants with home smoking policies in Scotland, including those based on informal discussions, to “negotiated” or “enforced” smoking restrictions [54]. A population based study by Germain and co-authors examined the responses of nonsmokers to smokers and SHS in Australia and found that many nonsmokers were unwilling to confront smokers, despite being bothered by SHS exposure [55]. Together, these studies suggest that responses to SHS policies and SHS management are mixed due to differences in social location and context, smoking status, and individual and personal characteristics and dynamics.

This project built upon this work by examining differential effects on women and men of varied income levels and smoking statuses, in experiences of SHS exposure and management (in both private and public spaces) and responses to smoking restrictions in Vancouver.

## 3. Methods

To explore the everyday experiences of women and men in relation to smoking, SHS, and SHS policies, we employed a variety of qualitative methods. Participants were recruited via advertisements in universities, coffee shops, hospitals, local media, and Craigslist (a free online classified advertisement). Participants who responded to advertisements completed a telephone screening to determine their eligibility for the study (exposed to SHS daily or almost daily and who were 19 years and older). After telephone interviews were completed with 40 individuals, additional participants were put on a waiting list to participate in focus groups.

Forty telephone interviews were held between March 2010 and February 2011, with 21 women and 19 men in Greater Vancouver. Women and men were also screened according to their income levels and classified as low income or not low income according to their self-reported combined family income before deductions, using the Low-Income Cut-Offs (LICOs) from Statistics Canada for 2004, and based on Vancouver population size (500,000+) (see Table 1). Interviews were held with 9 low-income women and 9 low-income men, and 12 non-low-income women and 10 non-low-income men. Although participants' smoking status was recorded, sampling was not performed based on smoking status. Please see Table 2 for demographic characteristics.

Following individual interviews, we held focus groups with women and men to explore emerging themes in more depth. Focus groups were held with one group of 3 low-income women, one group of 3 non-low-income men, and one non-low-income woman (additional participants were recruited but did not participate in the focus group). We were unable to recruit men living on a low income to attend a focus group. After providing consent, participants in phone interviews and focus groups completed a questionnaire (including demographics, smoking status and measures of exposure). Interviews and focus groups were semistructured and included questions about experiences of increasing SHS restrictions, how they deal with SHS, how SHS restrictions impact their own smoking, if and how they negotiate child-caring duties within the context of SHS and SHS restrictions, how they deal with partners, friends, and family within these contexts, how they experience the built environment, including public and private spaces, and their experience of the general public and their reaction to both smoking, smoke exposure and women and men's attempts to control SHS. The interviews were conducted by a trained female interviewer over the phone, and the focus groups by a trained, female facilitator in a meeting room at BC Women's Hospital (transportation vouchers and child care reimbursement were offered). Participants received gift cards to local retailers as honorarium for their participation, in the amount of $20 for the phone interviews and $40 for the focus groups.

All interviews and groups were recorded and transcribed, and qualitative analysis (NVivo 8) software was utilized to analyze interview and focus group transcripts. Recurring themes were identified, paying particular attention to gendered factors and differences between women and men, and income levels. Data associated with each specific theme were organized under each code. Preliminary themes were discussed and reviewed in a team meeting, and themes further refined, the key themes identified form the basis of this paper.

## 4. Results

Depending upon their particular experience, women and men described SHS exposure and challenges in managing smoke exposure in both public (in workplaces, bars and restaurants, outside of public buildings, and in beaches and parks) and private spaces (particularly the home, but also in cars). Examining SHS exposure more broadly, in addition to specific experiences of SHS policies in Vancouver (situated primarily in public spaces) allows for an exploration of the specific vulnerabilities that women and men of varied incomes and smoking statuses encounter, and the potential unintended consequences of SHS policies. Results are organized according to three key themes: (1) the reshuffling and relocation of where people are smoking; (2) SHS management and the impact on social relations and interactions; (3) disparities in the effect of policies and management of SHS.

### 4.1. Reshuffling and Relocating Where People Smoke

When asked about their experience of SHS policies, many participants reported being satisfied with smoking restrictions and felt their exposure had decreased as a result. However, other participants thought that policies have not decreased smoking or smoke exposure, but rather simply reshuffled where people are smoking.

They're (smokers) just finding other places to do it, that's all, and then people's exposure increases in different scenarios, like the bus stop or places where maybe they weren't or like a lot less so years ago when people could smoke under a building or whatever (female, non smoker, not low income).

With people no longer permitted to smoke in the workplace, restaurants, bars, hotels, some apartments/housing, and most recently beaches and parks, the spaces where smoking is allowed are shifting and narrowing. Participants clearly articulated a shift in social norms regarding smoking, connected to increasing restrictions on smoking locations. As it has become less socially acceptable to light up, smokers who abide by smoking restrictions must navigate their use of public space in new ways.

A smoker is going to smoke pretty much everywhere. But there'll be some of those smokers that are really aware that their smoking is, people do not like it, and you'll see them go into like a corner or something, like kind of out of the way, like they'll get up out of a restaurant and they won't like smoke right in front of the restaurant, because they know the windows are open, the doors are open, it's going to come inside. And so you'll see them either go across the street or you know around the corner, and they'll be somewhere where you know, that it's not going to affect somebody else. (male, non-smoker, not low income).

Some participants described how smokers are being moved to increasingly marginal spaces, such as street-corners or alleyways, spaces physically and visibly divided from the majority of nonsmokers. Further implications of the physical and social marginality were made by participants who compared a smoker to a “back alley drug user.”

Participants also commented on the increased concentration of smokers in certain public spaces such as at bus stops, sidewalks, outside of workplaces, restaurants, and bars. While smoking policies do exist for bus stops and business fronts in Vancouver, these restrictions are frequently broken or SHS travels to the area where others are positioned or waiting. As smokers reshuffle where they are smoking, or cluster in particular smoking areas, those people trying to manage SHS must also manoeuvre their environment in new ways. Expanding smoking restrictions therefore impact the use of space, and the social interactions between smokers and nonsmokers over SHS exposure.

Although SHS policies are focused primarily on public spaces, another effect of this reshuffling is the displacement of smoking into private spaces, particularly homes and cars where restrictions are less likely to exist. For example, one participant explained:

At my place, we have a condo that used to allow smoking on the balcony, and about six months ago they actually came out with a bylaw that said “No smoking on balconies.” So my girlfriend now smokes inside (male, non smoker, not low income).

Similarly, some female participants spoke about their male partner's preference to remain in the home (where he could continue to smoke) rather than visit restaurants or bars with smoking restrictions.

My husband won't go out for a nice dinner, because he thinks “I cannot have a cigarette, so I ain't going to no—you know, unless we can go somewhere fast food or whatever,” so a lot of times we just do not go out with him, you know. I'll go out on my own or go out with a friend or my daughter or whatever. (female, low income, former smoker)

With increasing prohibitions around public smoking and the movement of smoking into private spaces, individuals are increasingly required to negotiate smoking in this domain. In cases where smokers and nonsmokers are sharing a space, or where there are disagreements related to home smoking policies, the power differences between individuals (and partners in particular) may come to the fore. SHS policies are transforming the use of public and private space and the social interactions within these spaces.

### 4.2. SHS Management and the Impact on Social Relations and Interactions

Participants were asked to describe their experience of managing SHS and the impact on social relations and interactions. These experiences were influenced by tolerance to SHS and perceived priority of SHS management; place of exposure (private or public space) and interactions with smokers in that domain. 

#### 4.2.1. Tolerance to SHS & Priority of SHS Management

Some women and men reported being intolerant to smoking and smokers in their lives, choosing to limit their time with friends and family who smoke.

Well there's a couple of friends I used to go and just visit and have coffee and tea and that, but more and more it turned me off because every time I'd go I'd just reek of tobacco, and I couldn't handle it no more. Finally I told them too, right, and I said “Well it's not you, it's the tobacco” it just, you know … it was too much. So you avoid some people that it's, you know, and it's too much. (male, former smoker, not low income).

I tend to spend little time with people who smoke. I just have less and less tolerance for it. So people who were my friends and smoke, I just, I do not spend time with them anymore or if I have family members out of town who smoke, I won't stay at their home. (female, non-smoker, not low income).

Women and men from both income groups discussed choosing partners and friends who are nonsmokers and avoiding those who smoke, but this was more salient for non-low-income women and men. For these individuals, avoiding SHS is such a priority that they made changes to their social groups in an effort to limit exposure.

In contrast, some participants reported that they are not bothered by smoke and therefore have not made any changes to limit their smoke exposure. In some cases, participants implied that the value of their relationships (with friends, family, partners) outweighed their concerns over SHS exposure:

I do not [have a smoke-free home] If I had a smoke-free [home] nobody would come visit me! (female, non-smoker, low income).

Socially … honestly I cannot say I've done anything to decrease—like the friends, I'm not—the friends that I have I'm not going to drop, right. (male, smoker, low income).

Women and men from both low and non-low-income groups reported tolerance for SHS or no actions to manage SHS, but this feedback more often came from low-income women and men. These quotes (particularly the first quote) also indicate how the composition of the social group influences reactions to smoking restrictions and SHS management. If most or all individuals in a social group are smokers, reducing exposure may mean reducing or eliminating time spent with friends and family who smoke. Many participants did not want to reduce or give up social activities and relationships they share with people who smoke.

#### 4.2.2. Variations in Interactions over SHS Exposure in the Private Sphere

Often, the greatest and most sustained source of exposure to SHS came from friends and family members within private spaces (their home, car or during social gatherings with friends/family) rather than strangers in public spaces. For those people who have a partner or family member who smokes or are sharing a living space with someone who smokes, the effect of public smoking restrictions on decreasing overall SHS exposure may be negligible. Participants who experienced SHS exposure in the home and were trying to limit or reduce their exposure, discussed how they negotiated smoking restrictions in the home, or experienced challenges or conflict with friends or family over smoking in the home.

For example, the following nonsmoking participants explained how they have negotiated smokefree spaces in their homes and social environment to avoid exposure while still maintaining relationships.

[One] of my friends, he's kind of an addict to it. We're friends up to a point, but you know he does not smoke in front of me, he goes outside … he came to my place; I make it abundantly clear, if you want to smoke you go outside. And that's it, he knows the rules … in my case I won't bend on that. (male, non-smoker, not low income).

Over my environment I [have control]. If someone comes over to my house and wants to smoke, you know, it's kind of my house, my rules, but I do not have that control over other people's environments so it's a lot harder (female, non-smoker, not low income).

All respondents suggested that they negotiate smoke-free spaces with friends, family, and partners, yet non-low-income participants more often described the process of negotiating a smoke-free space. These participants implied that they have the means to negotiate rules around smoking, particularly in their homes or cars. In addition, some smokers revealed ways in which they cooperate in either reducing their smoking around others or their partner.

I think—rules or not, if you're respectful of the people that you have relationships with, then you take their feelings into consideration when it comes to something like smoking. I do not apologize to anybody for the fact that I do smoke cigarettes, but I'm very respectful of their wishes. (female, smoker, not low income).

In some cases, agreements are made between smokers and nonsmokers, and smokers may also willingly accommodate the requests or “rules” of nonsmokers. For these participants, there is a sense of cooperative exchange between smokers and nonsmokers.

Yet for other participants, managing SHS exposure in the home was marked by conflict with partners, friends, or family members.

The only thing I find is that with my husband, a lot of people won't come and visit because of his smoking. And you know, he's really stubborn when it comes to trying to tell him that he cannot smoke inside a house, right. (female, non smoker low income).

P: [I'm] fighting with my mom and my dad all the time.

I: What's the gist of the fight? Like are you trying to tell them to stop, or you're trying to tell them to smoke someplace else, or?P: Smoke someplace else, do not waste our money, I work hard, you smell, I do not want to kiss you, I do not want to touch you, you stink, it goes on. (female, smoking status unknown, low income).

Women and men from low-income and non-low-income groups reported arguing with partners or family members over their smoking and some participants indicated that smoking had resulted in a previous break-up with a partner. However, women, and particularly women living on a low income, were more likely to cite challenges in negotiating a smoking restriction in the home. Differences in smoking status, tolerance to SHS, and decision-making power contribute to the interpersonal challenges and conflict associated with the management of SHS.

#### 4.2.3. Interactions over SHS Exposure in the Public Sphere

For some individuals, the main source of SHS exposure is from strangers, mostly in public outdoor spaces. Participants who reported having strict no-smoking policies within their homes and cars often discussed challenges in managing their SHS exposure in outdoor public spaces. In attempting to limit their smoke exposure in these environments, participants described avoidance strategies, or confrontation and in some cases conflict with smokers.

For example, women and men from both income groups discussed how they reposition themselves in public spaces, avoid particular activities (smoky clubs or bars), or change their commuting route to avoid smokers.

Sometimes what I do is I try to like I put my hands up and kind of air out the air in front of my face, or sometimes I'll just walk fast and try to avoid it, or I'll move myself away, try to move myself away as much from the area (female, non smoker, low income).

I mean aside from standing upwind I'm always, you know, juggling myself around so I'm out of it. (male, former smoker, not low income).

Conscious repositioning allowed participants to create and maintain distance from SHS and avoid contact with smoke and smokers.

Similarly, some participants described how they alter their smoking behaviour to avoid affecting nonsmokers. However, other participants reported difficulties in managing their smoke exposure in public spaces, sometimes escalating into conflict when confronting strangers who were not respecting SHS policies.

I went to the Salvation Army for a meal, you know, so a guy comes and sits beside me, picks up a cigarette and starts blowing smoke at me, you know. And then I asked him, you know “That's kind of rude” and he says “Oh well, this is the way people smoke”, you know, right. “I'm allowed to smoke at this table here!” you know “Okay”. But you know, I just got up and left. (male, non smoker, low income).

I've had one where the guy came back and I was sitting there waiting in a line-up to buy tickets and I asked him politely. He looked at me and said “Get lost” and “moron” and this and that and started mouthing off to me. (male, former smoker, not low income).

These statements display the tension that exists between some smokers and nonsmokers. Several participants described smokers as being “rude” or “inconsiderate” in subjecting them to SHS and were hostile when confronted about their smoking. However, variations clearly exist in how nonsmokers cope with and control their exposure, and the interaction that occurs between smokers and nonsmokers.

### 4.3. Disparities in the Effect of Policies and Management of SHS

Participants suggested that not all women and men are experiencing the potential benefits of SHS policies (i.e., reduced SHS exposure, improved health). Gender roles and responsibilities, and social and economic differences impact women and men's vulnerabilities to smoking and SHS exposure. An unintended and undesirable consequence of smoking restrictions is the potential to reinforce or enhance these vulnerabilities, contributing to disparities in health between women and men, and subpopulations of women and men. We have identified the potential for the following disparities related to SHS exposure and management: stigma during pregnancy and parenting, gender differences in vulnerabilities to exposure in the home and workplace, gender differences in the management of SHS and socioeconomic disadvantage. 

#### 4.3.1. Stigma during Pregnancy and Parenting

Both men and women spoke about their experiences of stigma or discrimination as smokers. However, one of the key vulnerabilities that emerged for women in regards to SHS is the effects of the denormalization of smoking particularly during pregnancy and mothering, and the heightened potential for stigma and shaming within this context. The following quotes demonstrate the strong pressures that exist in regards to smoking during pregnancy:

P: … I have not been friends with anyone that smokes when they're pregnant, and in this day and age I do not know if I could be friends with them.

I: It's a really, it's a contentious issue, right. Like so what happens if you live with that woman who's pregnant, right?

P: If I was a man, I would probably say “I'm going to divorce you if you do not, and I'm going to fight for the child.” Yeah, I would divorce someone for that. (female, smoking status unknown, low income).

[My partner] knows she cannot, she's pretty, she's aware that she cannot, you know, endanger another person or a child or a life, so I know for a fact that if she was to get pregnant she wouldn't be smoking, I know that. She would never put a child at risk like that. (male, non smoker, not low income).

During interviews and focus groups, smoking during pregnancy was framed as an irresponsible behaviour. Media and health advocacy around SHS have often focused on exposure during pregnancy and among children. For women who are not able to spontaneously quit, the moral implications associated with smoking during pregnancy and parenthood exacerbates the feeling of stigma and shame for women, hindering their capacity to reduce or quit smoking. An unintended consequence is that women may avoid seeking cessation help from practitioners or their partners or families for fear of conflict, judgement, or incrimination.

#### 4.3.2. Gendered Aspects of Vulnerability to Exposure in the Home and Workplace

Women more often talked about their challenges in managing their smoke exposure in the home. Higher rates of smoking among men were understood to result in greater rates of exposure for women in the home if they have a male partner who smokes. Furthermore, participants articulated experiences of conflict related to SHS as stemming from gender differences in power or control over financial resources. For example, the following female participants suggested that they lack decision-making power in the home.

I: Okay. Now can you describe any challenges you have in managing your smoke exposure?

P: Yeah, I like having air in my house (female, non smoker, low income).

Well the way it is right now, I mean my husband's the one that smokes, so he does not, like “Oh no, eh, whoever wants to come and smoke, eh, why not?” Excuse me. That's all, I mean this—it's almost gotten us for, you know, a divorce over this issue. (female, smoking status unknown, low income).

In particular, the ownership of private space (homes, cars) was understood to warrant the decision-making authority around smoking or smoking restrictions. Due to sex segregation of both paid and domestic work, women may have more responsibilities within the domestic sphere but a limited ability to participate in decision making on SHS policies. With increasing public smoking restrictions, a potential unintended consequence is that women may endure more SHS exposure and challenges related to SHS management in the home.

While women face particular vulnerabilities in the home, participants thought that men were more likely to be exposed to SHS in the workplace. In particular, men involved in trades-based occupations, which tend to be male dominated, were perceived to be more vulnerable to smoking and smoke exposure. These types of occupations may in certain cases be exempt from workplace SHS laws. For example, although smoking in work vehicles is prohibited in British Columbia, participants still reported observing or experiencing male workers smoking during their commute to work sites. Similarly, if work is done outdoors, employees may be permitted to smoke on the job site. Gender divisions in the home and workplace shape women and men's vulnerabilities to smoking and smoke exposure.

#### 4.3.3. Gendered Aspects of the Management of SHS

Gender differences were noted in responses to, and management of, SHS. Findings suggest that men confront a particular set of challenges in managing SHS. According to feedback from participants, men face more pressure to smoke and less social support to assist with reduction or cessation.

There might be more peer pressure to smoke with men, because it's, I do not know, like from the crowd that I come from, it's not really like okay for the women to smoke, but the men typically smoke all the time and because my boyfriend does not smoke, so when he comes over my father is like “Oh, here a smoke, have a smoke,” and my boyfriend's like “Oh no, that's fine.” And then it's like “No, no, no, no, here have one.” So it's, it's just a little awkward, so I guess a man thing I guess. (female, smoker, not low income).

In some situations, men may be encouraged by other men to smoke, possibly due to expectations regarding “masculinity” or the use of smoking as a socialization tool. While women may be more likely to be exposed to their partner's smoking, men may face unique vulnerabilities to SHS due to their gender identities and social expectations regarding smoking and tolerance to SHS.

[Secondhand smoke is] not as that much of an issue for us [men], like it is on women. Like men it's not that big of a deal … [Have] a smoke around [a woman], she'll cover her nose, she'll pull her shirt over her nose, and she goes “Can you stop doing that? Can you smoke over, go down the street,” like she'll actually freak out, she hates it. And like some of the guys, like they just do not do anything. They just stand there, whatever. The girls always are just like running around and like, trying to like get away from it. (male, smoker, low income).

Yeah. I think that men generally, when they're exposed, they—my impression is that they just put up with it, they do not really, or it does not seem to bother them as much … Just because, well, I do not know, to me it's perceived like a macho thing and you know, like they seem to be just more compliant or more acceptable of it. (female, non-smoker, low income).

It may be more socially acceptable for men to smoke compared to women and more acceptable for women to ask others not to smoke. While women may have less power or capacity to make decisions regarding smoke exposure in partnerships (particularly in the home), in certain contexts women may be more likely to ask others not to smoke or demonstrate less tolerance for smoke exposure.

Well, I would have to say that the men that I know are less likely to complain to friends who happen to be smokers, and are more likely to put up with it. Women possibly who are in relationships may not be as likely to complain to the partner that they happen to be with, but that's just an assumption. (female, non-smoker, low income).

In some social circumstances, it is not perceived as “manly” to voice concern or “complain” about SHS, particularly within groups of men. Women, on the other hand, were regarded as being more health conscious and more likely to limit their exposure to SHS. However, under different conditions, participants thought that women would be less assertive and less likely to confront a stranger about their SHS.

I guess if it were a group of men smoking and a woman being exposed to it, she may feel sort of, either a little bit more daunted if she were going to approach them about it or ask them to move away, or something like that, opposed to a guy asking a group of women. (female, non-smoker, not low income).

It may not be that either women or men are more at risk for SHS exposure, but rather that they encounter unique vulnerabilities based on gender roles and expectations, and power differences in relationships or social situations.

#### 4.3.4. Socioeconomic Disadvantage

The majority of participants thought that people living on a low income would be more vulnerable to SHS, face more smoking-related challenges and be less likely to benefit from SHS policies.

[People living on a low income] would have more challenges in life, so it could be a coping mechanism, or someone in their family is the one smoking so there would be even greater chances of being exposed to second-hand smoke … They may not be able to afford other options that someone more well off could if they wanted to choose a healthier lifestyle (female, non smoker, not low income).

Participants noted that smokers tend to be poor and have fewer resources to afford healthier options, experience more stress and anxiety and are more likely to use smoking as a coping mechanism. Some people living on a low income use smoking to cope with mental illness, and therefore face more barriers to reducing or quitting smoking.

Participants thought that people living on a low income tend to be surrounded by more smokers, and also that smoking restrictions are less likely to be regulated. Low-income neighbourhoods or housing areas often lack access to private outdoor space, creating challenges for those individuals trying to reduce their smoking or SHS exposure.

Um, I would feel that if you're in a lower income area for example, living-wise, you are kind of grouped together in a smaller area and more pushed together, I guess, and it's just a smaller space with more people smoking, so it would be harder to get away from (female, smoker, low income).

Women and men living on a low income are more likely to live in more crowded areas, with more smokers and less safe, open spaces. These physical constraints limit opportunities to avoid SHS exposure in spite of increasing restrictions. The physical, social, and economic barriers low-income women and men encounter to reducing smoking and smoke exposure may reinforce or intensify health-related disparities.

## 5. Discussion

Smoking restrictions have resulted in a reshaping of both the social and physical environment. Participants described a reshuffling and relocating of where people are smoking, bringing new challenges both for smokers and for those managing smoke exposure. These findings align with previous qualitative work done by Bell and colleagues that found that smokers in Vancouver are experiencing a narrowing of space due to location restrictions [50]. A study by Kaufman and colleagues [56] in Toronto also revealed how nonsmokers are navigating their environment in new ways to avoid smoking in outdoor urban spaces, particularly around doorways of businesses where smoking continues to cluster. 

Likewise, participants in our study discussed difficulties in avoiding outdoor SHS, particularly in bus stops and near businesses. While smoking is prohibited in these spaces in Vancouver, the lack of other available spaces for smoking in public, coupled with the difficulty of enforcement in all places at all times, impedes the effect of policies in these spaces. Without the effective enforcement of smoking restrictions in public outdoor spaces, the narrowing of available space for smokers, along with the increasing denormalization of smoking, may increase the presence and frequency of social tensions between smokers and nonsmokers. Smoking restrictions need to be coupled with strong and tailored tobacco reduction and cessation support, particularly for vulnerable populations who experience more barriers to quitting.

There was variation in participants' management of SHS and the impact of restrictions on social relations and interactions. The differences identified by participants in the effects of SHS policies and SHS management on social interactions include the attitudes towards and tolerance of smoking and SHS, relationship dynamics, place of exposure, and capacity or power to control exposure. Smoking restrictions appear to be contributing to the increasing division of smokers and nonsmokers and are impacting social exchanges related to SHS. But differences in tolerance to SHS and/or power differences structure some participants' ability to modify their SHS, sometimes leading to conflict in the private domain [57].

The differences between participants' experiences of policies and SHS management are backed up by other qualitative studies that have identified different levels of tolerance, interaction styles, and power dynamics related to smoke exposure [53, 54], the implementation of home smoking policies [54], and reducing or quitting smoking during pregnancy [58]. Poland and colleagues identified heterogeneity in smokers and nonsmokers attitudes and responses to SHS. They distinguish between “reluctant” smokers who demonstrate concern over smoking around others, “easygoing” smokers who support restrictions and limit their smoking around others, and “adamant smokers” who are less inclined to limit their smoking around nonsmokers [53]. Similarly, they identified nonsmokers as either “adamant” or highly intolerant to smoking, “unempowered” smokers who oppose but do not or cannot manage their exposure, or “laissez-faire” nonsmokers who are less opposed and therefore less likely to manage their exposure. Different relationship dynamics related to the implementation of a home SHS policy, ranging from voluntary to negotiated or enforced restrictions have also been described [54]. Finally, Bottorff and colleagues have discussed the social context and relationship dynamics associated with smoking reduction and cessation, within the context of pregnancy. They found complex tobacco-related interaction patterns between couples when quitting during pregnancy [58], including disengaged (individualized decision-making), conflictual (shaming, monitoring, hostility), and accommodating (work together/open communication) interaction patterns. For example, for couples with a conflictual interaction pattern, smoking cessation during pregnancy may result in the “policing” of the other partner's smoking behaviour [58] or even abusive and controlling behaviour [57]. Together with our findings, these suggest that SHS management is a complex process, influenced by individual tolerance of SHS and responses to smoking restrictions, along with social and situational differences and interpersonal dynamics and relationships. Tobacco control policies and programs are needed that respond to these complexities and support varied approaches to reduction, cessation, and SHS management.

While the process of denormalization may in fact encourage some people to quit smoking, for smokers who are unable or lack the resources to quit, smoking-related stigma negatively impacts their health and quality of life and may undermine their ability to quit. Individuals who experience social stigma may be more likely to conceal their smoking, inhibiting access to cessation support from health care providers and friends and family [59]. In particular, our study revealed high levels of denormalization and potential for smoking-related stigma in the context of pregnancy and motherhood. Policies aimed at reducing SHS have also been found to contribute to smoking stigmatization among mothers by others, and that these effects are particularly harmful for socially disadvantaged mothers [60]. Given that women who smoke during pregnancy are more likely to be socially disadvantaged and experience more barriers to quitting, these findings support the need for women-centred approaches to smoking cessation and reduction during pregnancy that reduce stigma and focus on the health of women in and of itself [61]. Emerging qualitative research reveals that new fathers who smoke experience negative judgement, and perceive smoking to conflict with dominant ideals of masculinity and their role as provider and protector of the family [62]. While this theme did not emerge during interviews and focus groups in our study, this remains an important area for further research.

Participants described unique vulnerabilities related to power differentials, access to economic resources and gendered roles and relations. Our findings suggest that women have more challenges in reducing smoke exposure in the home and men in the workplace. In support of this, a study by Paul and colleagues found that low-income men working outdoors were vulnerable to smoking and smoke exposure [13]. While participants in our study thought men are more exposed to SHS at work, other research suggests that women may also face special challenges in managing SHS in the workplace. For example, research reveals that women more often occupy restaurant and bar jobs and face challenges in enforcing smoke-free establishments in these settings [63] and also encounter a prosmoking context when working in office and retail positions [13]. Together, these findings suggest that both men and women are at risk of SHS exposure at work, in part due to a gendered and classed division of labour.

The finding that women tend to cite more difficulties in negotiating a smoke-free home may in part be due to higher smoking rates among men, making it more likely that a woman would be exposed to a male partner's smoking. Furthermore, research reveals that as smoking restrictions intensify, smoking may shift into the home, valued by some smokers as one of the last “comfortable” places to smoke [13]. Yet these differences are also indicative of power differences that exist between women and men, particularly related to the control of financial resources and home ownership. Men more often control the financial resources in the home, and women may be less able to speak up about SHS exposure. Given that women and children living with smokers are at an increased risk of disease and death, these reports have serious health implications [64]. Smoking cessation interventions are needed for women that explicitly address these factors and differences in power and incorporate negotiation skills and empowerment principles.

On the other hand, our findings indicate that men encounter specific vulnerabilities to SHS due to perceptions of masculinity. Smoking among men appears to be more socially acceptable, and asking others not to smoke conflicts with social understandings of “manliness”. For example, a study by Germain and colleagues found that males were less likely than females to move away from SHS [55]. Smoking may also be used as a tool for socializing, in the absence of other opportunities for meaningful connections between men. Morrow and colleagues discuss how the practice of risk behaviours, such as smoking, by men are used as an expression of masculinity [65]. Other research found that men hesitate to use cessation resources due to dominant ideals of masculinity such as “independence” and “strength” [66]. Findings from our study support similar dominant ideals of masculinity that prevent men from limiting their exposure to SHS. Gender-specific and gender-sensitive policies and smoking cessation interventions are needed that account for and address these differences in a men-centred manner.

Furthermore, women and men living on a low income experience numerous barriers to smoking reduction and cessation and SHS management. Smoking in low-income areas may be normalized, smoking restrictions less enforced, and individuals experiencing the many stresses associated with living on a low income may find it difficult to quit [13, 49]. These findings support previous research revealing that people living on a low income are more likely to inhabit a “prosmoking context,” receive less education on the health risks of smoking and SHS exposure and are less likely to have smoke-free homes [13]. While there may be less stigma associated with smoking in low-income areas, the relative normalization of smoking within low-income areas makes reducing or quitting smoking or managing smoke exposure a greater challenge. Christakis and Fowler found that the presence of smokers in one's social network discourages smoking cessation, as smokers tend to group together and in effect normalize smoking within the social group [67]. In a similar vein, the combined stigmatization of smokers with the spatial segregation of low-income groups has been described as producing “smoking islands” that encourage continued smoking and impede cessation efforts [49]. It has been suggested that the perception of smoking as socially unacceptable and the stigmatization of smokers is facilitated by the shift in tobacco consumption to low-income groups [42]. Tobacco control initiatives are required that acknowledge and respond to these inequities and prevent further stigmatization, by addressing the social determinants of health in tailored smoking reduction and cessation interventions. 

### 5.1. Limitations

There are a number of methodological limitations to report. First, due to the qualitative nature of this study, we cannot draw conclusions about the observed differences between women and men of varied income levels in British Columbia or Canada, in general. Second, we were unable to recruit men living on a low income to attend a focus group, and only met with one non-low-income women for a focus group (more women were recruited, but did not show up to the focus group). Because of this, we were only able to collect rich focus group data from low-income women and non-low-income men. Third, although participants' smoking statuses were recorded, sampling was not performed based on smoking status. We recruited more nonsmokers (25) than smokers (15) for the telephone interviews. Therefore, the data collected and presented here does not capture the experiences and viewpoints of smokers and nonsmokers equally. Finally, focus group participants were not identified in connection with questionnaire data during the audio recording or on transcripts. Because focus groups were not organized according to the smoking status of participants, we are therefore unable to include smoking statuses for the quotes of some focus group participants. Despite these limitations, this qualitative study surfaced important themes related to the experience of smoking restrictions and SHS management for women and men of varied income levels and smoking statuses, that warrant further research and consideration during policy development.

## 6. Conclusions

The experience of smoking restrictions and the management of SHS is influenced by the social context (relationship with a partner, family member or stranger, and control of resources), the space of exposure (outdoor/public or private space, worksite), the social location of individuals involved (gender, income), and tolerance to SHS. As smoking restrictions increase, both smokers and nonsmokers are required to develop new skills to navigate the built and social environment in new ways. Tobacco control policies and interventions are required that acknowledge and respond to the specific vulnerabilities of women and men, and low-income subpopulations. Approaches are needed that prevent further marginalization of the groups most vulnerable to smoking and SHS smoke exposure, such as low-income women, while maximizing the effect and impact of policies. For example, these may include health education messaging that is gender and diversity sensitive, gender-specific workplace interventions, the training of health care providers to address and respond to stigma, and the integration of tobacco control policies with economic and social policies (including housing, child care, and antiviolence). Policies need to be gender sensitive and tailored with women and men in mind, to target (and measure) the unique issues that women and men, and subpopulations of women and men, encounter when managing SHS.

## Figures and Tables

**Table 1:** Income classification scheme^1^.

Before-Tax Low-Income Cut-Offs (LICOs), 2004	
Family size	Population of community of residence
	500,000 +

1	$20,337
2	$25,319
3	$31,126
4	$37,791
5	$42,862
6	$48,341
7 +	$53,821

^1^Notes: this table uses the 1992 base. Income refers to total pretax household income.

Source: prepared by the Canadian Council on Social Development using Statistics Canada's Low Income Cut-Offs, from Low income cut-offs for 2004 and low income measures for 2002 Catalogue #75F0002MIE2005003.

**Table 2:** Demographic characteristics of telephone interview participants.

	Smokers	Nonsmokers	Total
Group 1= male, low-income	4	5	9
Group 2= male, not low-income	3	7	10
Group 3= female, low-income	4	5	9
Group 4= female, not low-income	4	8	12
Total	15	25	

## References

[1] Non-smokers Rights Association, Canada.

[2] Asbridge M (2004). Public place restrictions on smoking in Canada: assessing the role of the state, media, science and public health advocacy. *Social Science and Medicine*.

[3] Department of Health & Human Services US The health consequences of involuntary exposure to tobacco smoke: a report of the Surgeon General.

[4] Flouris AD, Metsios GS, Carrillo AE (2009). Acute and short-term effects of secondhand smoke on lung function and cytokine production. *American Journal of Respiratory and Critical Care Medicine*.

[5] Callinan JE, Clarke A, Doherty K, Kelleher C (2010). Legislative smoking bans for reducing secondhand smoke exposure, smoking prevalence and tobacco consumption. *Cochrane Database of Systematic Reviews (Online)*.

[6] Pickett MS, Schober SE, Brody DJ, Curtin LR, Giovino GA (2006). Smoke-free laws and secondhand smoke exposure in US non-smoking adults, 1999–2002. *Tobacco Control*.

[7] Naiman AB, Glazier RH, Moineddin R (2011). Is there an impact of public smoking bans on self-reported smoking status and exposure to secondhand smoke?. *BMC Public Health*.

[8] Grassi MC, Enea D, Ferketich AK, Lu B, Nencini P (2009). A smoking ban in public places increases the efficacy of bupropion and counseling on cessation outcomes at 1 year. *Nicotine and Tobacco Research*.

[9] Hackshaw L, McEwen A, West R, Bauld L (2010). Quit attempts in response to smoke-free legislation in England. *Tobacco Control*.

[10] Hahn EJ, Rayens MK, Langley RE, Darville A, Dignan M (2009). Time since smoke-free law and smoking cessation behaviors. *Nicotine and Tobacco Research*.

[11] Lemstra M, Neudorf C, Opondo J (2008). Implications of a public smoking ban. *Canadian Journal of Public Health*.

[12] Greaves L, Jategaonkar N (2006). Tobacco policies and vulnerable girls and women: toward a framework for gender sensitive policy development. *Journal of Epidemiology and Community Health*.

[13] Paul CL, Ross S, Bryant J, Hill W, Bonevski B, Keevy N (2010). The social context of smoking: a qualitative study comparing smokers of high versus low socioeconomic position. *BMC Public Health*.

[14] Moore RS, Annechino RM, Lee JP (2009). Unintended consequences of smoke-free bar policies for low-SES women in three california counties. *American Journal of Preventive Medicine*.

[15] Datta GD, Subramanian SV, Colditz GA, Kawachi I, Palmer JR, Rosenberg L (2006). Individual, neighborhood, and state-level predictors of smoking among US Black women: a multilevel analysis. *Social Science and Medicine*.

[16] Diez Roux AV, Merkin SS, Hannan P, Jacobs DR, Kiefe CI (2003). Area characteristics, individual-level socioeconomic indicators, and smoking in young adults: the coronary artery disease risk development in young adults study. *American Journal of Epidemiology*.

[17] Miles R (2006). Neighborhood disorder and smoking: findings of a European urban survey. *Social Science and Medicine*.

[18] Greaves LJ, Hemsing NJ (2009). Sex, gender, and secondhand smoke policies. Implications for disadvantaged women. *American Journal of Preventive Medicine*.

[19] Bauer JE, Hyland A, Li Q, Steger C, Cummings KM (2005). A longitudinal assessment of the impact of smoke-free worksite policies on tobacco use. *American Journal of Public Health*.

[20] Allwright S, Paul G, Greiner B (2005). Legislation for smoke-free workplaces and health of bar workers in Ireland: before and after study. *British Medical Journal*.

[21] Apollonio DE, Bero LA (2009). Evidence and argument in policymaking: development of workplace smoking legislation. *BMC Public Health*.

[22] Brander P Evaluation of the smoke-free environments legislation affecting workplaces.

[23] Fichtenberg CM, Glantz SA (2002). Effect of smoke-free workplaces on smoking behaviour: systematic review. *British Medical Journal*.

[24] Fong GT, Hyland A, Borland R (2006). Reductions in tobacco smoke pollution and increases in support for smoke-free public places following the implementation of comprehensive smoke-free workplace legislation in the Republic of Ireland: findings from the ITC Ireland/UK Survey. *Tobacco Control*.

[25] Houle B, Siegel M (2009). Smoker-free workplace policies: developing a model of public health consequences of workplace policies barring employment to smokers. *Tobacco Control*.

[26] Jaakkola MS, Jaakkola JJK (2006). Impact of smoke-free workplace legislation on exposures and health: possibilities for prevention. *European Respiratory Journal*.

[27] Nagelhout GE, Willemsen MC, de Vries H (2011). The population impact of smoke-free workplace and hospitality industry legislation on smoking behaviour. Findings from a national population survey. *Addiction*.

[28] Shavers VL, Fagan P, Alexander LAJ, Clayton R, Doucet J, Baezconde-Garbanati L (2006). Workplace and home smoking restrictions and racial/ethnic variation in the prevalence and intensity of current cigarette smoking among women by poverty status, TUS-CPS 1998-1999 and 2001-2002. *Journal of Epidemiology and Community Health*.

[29] Hyland A, Higbee C, Travers MJ (2009). Smoke-free homes and smoking cessation and relapse in a longitudinal population of adults. *Nicotine and Tobacco Research*.

[30] Mills AI, Messer K, Gilpin EA, Pierce JP (2009). The effect of smoke-free homes on adult smoking behavior: a review. *Nicotine and Tobacco Research*.

[31] Reace J (1994). Risk management of passive smoking at work and at home. *Saint Louis University Public Law Review*.

[32] Shopland DR, Anderson CM, Burns DM (2006). Association between home smoking restrictions and changes in smoking behaviour among employed women. *Journal of Epidemiology and Community Health*.

[33] Wakefield MA, Chaloupka FJ, Kaufman NJ, Orleans CT, Barker DC, Ruel EE (2000). Effect of restrictions on smoking at home, at school, and in public places on teenage smoking: cross sectional study. *British Medical Journal*.

[34] Tang H, Cowling DW, Lloyd JC (2003). Changes of attitudes and patronage behaviors in response to a smoke-free bar law. *American Journal of Public Health*.

[35] Brooks DR, Mucci LA (2001). Support for smoke-free restaurants among Massachusetts adults, 1992–1999. *American Journal of Public Health*.

[36] Hyland A, Higbee C, Borland R (2009). Attitudes and beliefs about secondhand smoke and smoke-free policies in four countries: findings from the international tobacco control four country survey. *Nicotine and Tobacco Research*.

[37] Yong HH, Foong K, Borland R (2010). Support for and reported compliance among smokers with smoke-free policies in air-conditioned hospitality venues in Malaysia and Thailand: findings from the international tobacco control Southeast Asia survey. *Asia-Pacific Journal of Public Health*.

[38] Bayer R, Colgrove J (2002). Science, politics, and ideology in the campaign against environmental tobacco smoke. *American Journal of Public Health*.

[39] Bell K, Salmon A, Bowers M, Bell J, McCullough L (2010). Smoking, stigma and tobacco ’denormalization’: further reflections on the use of stigma as a public health tool. A commentary on Social Science & Medicine’s Stigma, Prejudice, Discrimination and Health Special Issue (67: 3). *Social Science and Medicine*.

[40] Chapman S, Freeman B (2008). Markers of the denormalisation of smoking and the tobacco industry. *Tobacco Control*.

[41] Bayer R (2008). Stigma and the ethics of public health: not can we but should we. *Social Science and Medicine*.

[42] Bayer R, Stuber J (2006). Tobacco control, stigma, and public health: rethinking the relations. *American Journal of Public Health*.

[43] Thomson G, Wilson N, Edwards R, Woodward A (2008). Should smoking in outside public spaces be banned? Yes. *BMJ*.

[44] Repace J (2000). Banning outdoor smoking is scientifically justifiable. *Tobacco Control*.

[45] Chapman S (2000). Banning smoking outdoors is seldom ethically justifiable. *Tobacco control*.

[46] Chapman S (2008). Should smoking in outside public spaces be banned? No. *BMJ*.

[47] Bell K (2011). Legislating abjection? Secondhand smoke, tobacco control policy and the public's health. *Critical Public Health*.

[48] Siegel M (2007). Is the tobacco control movement misrepresenting the acute cardiovascular health effects of secondhand smoke exposure? An analysis of the scientific evidence and commentary on the implications for tobacco control and public health practice. *Epidemiologic Perspectives and Innovations*.

[49] Thompson L, Pearce J, Barnett JR (2007). Moralising geographies: stigma, smoking islands and responsible subjects. *Area*.

[50] Bell K, McCullough L, Salmon A, Bell J (2010). ’Every space is claimed’: smokers’ experiences of tobacco denormalisation. *Sociology of Health and Illness*.

[51] Ritchie D, Amos A, Martin C (2010). ‘But it just has that sort of feel about it, a leper’-Stigma, smoke-free legislation and public health. *Nicotine and Tobacco Research*.

[52] Ritchie D, Amos A, Martin C (2010). Public places after smoke-free—a qualitative exploration of the changes in smoking behaviour. *Health and Place*.

[53] Poland BD, Cohen JE, Ashley MJ (2000). Heterogeneity among smokers and non-smokers in attitudes and behaviour regarding smoking and smoking restrictions. *Tobacco Control*.

[54] Robinson J, Ritchie D, Amos A, Greaves L, Cunningham-Burley S (2011). Volunteered, negotiated, enforced: family politics and the regulation of home smoking. *Sociology of Health and Illness*.

[55] Germain D, Wakefield M, Durkin S (2007). Non-smokers’ responses when smokers light up: a population-based study. *Preventive Medicine*.

[56] Kaufman P, Griffin K, Cohen J, Perkins N, Ferrence R (2010). Smoking in urban outdoor public places: Behaviour, experiences, and implications for public health. *Health & Place*.

[57] Greaves L, Kalaw C, Bottorff JL (2007). Case studies of power and control related to tobacco use during pregnancy. *Women’s Health Issues*.

[58] Bottorff JL, Kalaw C, Johnson JL, Stewart M, Greaves L, Carey J (2006). Couple dynamics during women’s tobacco reduction in pregnancy and postpartum. *Nicotine and Tobacco Research*.

[59] Stuber J, Galea S, Link BG (2009). Stigma and smoking: the consequences of our good intentions. *Social Service Review*.

[60] Burgess DJ, Fu SS, van Ryn M (2009). Potential unintended consequences of tobacco-control policies on mothers who smoke. A review of the literature. *American Journal of Preventive Medicine*.

[61] Greaves L (2011). *Expecting to Quit: A best-practices review of smoking cessation interventions for pregnant and post-partum women*.

[62] Greaves L, Oliffe JL, Ponic P, Kelly MT, Bottorff JL (2010). Unclean fathers, responsible men: smoking, stigma and fatherhood. *Health Sociology Review*.

[63] Moore RS, Lee JP, Antin TMJ, Martin SE (2006). Tobacco free workplace policies and low socioeconomic status female bartenders in San Francisco. *Journal of Epidemiology and Community Health*.

[64] Wipfli H, Avila-Tang E, Navas-Acien A (2008). Secondhand smoke exposure among women and children: evidence from 31 countries. *American Journal of Public Health*.

[65] Morrow M, Barraclough S (2010). Gender equity and tobacco control: bringing masculinity into focus. *Global health promotion*.

[66] Bottorff JL, Oliffe J, Kalaw C, Carey J, Mroz L (2006). Men’s constructions of smoking in the context of women’s tobacco reduction during pregnancy and postpartum. *Social Science and Medicine*.

[67] Christakis NA, Fowler JH (2008). The collective dynamics of smoking in a large social network. *New England Journal of Medicine*.

